# Exploring the Factor Structure of Neurocognitive Measures in Older Individuals

**DOI:** 10.1371/journal.pone.0124229

**Published:** 2015-04-16

**Authors:** Nadine Correia Santos, Patrício Soares Costa, Liliana Amorim, Pedro Silva Moreira, Pedro Cunha, Jorge Cotter, Nuno Sousa

**Affiliations:** 1 Life and Health Sciences Research Institute (ICVS), School of Health Sciences, University of Minho, Braga, Portugal; 2 ICVS/3B’s, PT Government Associate Laboratory, Braga/Guimarães, Portugal; 3 Centro Hospitalar do Alto Ave—EPE, Guimarães, Portugal; Texas Tech University Health Science Centers, UNITED STATES

## Abstract

Here we focus on factor analysis from a best practices point of view, by investigating the factor structure of neuropsychological tests and using the results obtained to illustrate on choosing a reasonable solution. The sample (n=1051 individuals) was randomly divided into two groups: one for exploratory factor analysis (EFA) and principal component analysis (PCA), to investigate the number of factors underlying the neurocognitive variables; the second to test the “best fit” model via confirmatory factor analysis (CFA). For the exploratory step, three extraction (maximum likelihood, principal axis factoring and principal components) and two rotation (orthogonal and oblique) methods were used. The analysis methodology allowed exploring how different cognitive/psychological tests correlated/discriminated between dimensions, indicating that to capture latent structures in similar sample sizes and measures, with approximately normal data distribution, reflective models with *oblimin* rotation might prove the most adequate.

## Introduction

Factor analysis, which was first introduced to analyze data from large numbers of psychological tests [1], is an important technique that provides a means of data reduction to obtain an “orderly” simplification from a group of interrelated measures. It assumes that some variables of theoretical interest cannot be observed directly, but rather it is by exploring (modeling) relationships between the measurable/observable variables that information can be obtained on the underlying smaller number of unobserved variables (also called latent variables or factors) [2]. There are two basic types of factor analysis: exploratory factor analysis (EFA) and confirmatory factor analysis (CFA), with their applicability being based on the tests’ intrinsic differences. EFA is at its core a “data-driven” (*a posteriori*) approach, while CFA is “theory-driven” (*a priori*) [3, 4]. In broad terms, EFA and CFA constitute two discrete classes of factor analysis aimed to use a common factor model to represent relationships between observed variables, with a minimum number of factors. EFA aims to explore data and to provide information regarding the number of factors that best fit to the data, with each factor constituting a latent variable that underlies different variables. Although it is not always a means of testing the hypothesis, there are hypothesis tests of the resulting factor solution from EFA (for example, in the maximum likelihood method there is a goodness-of-fit test, the chi-square statistics). In the presence of a theory, the use of CFA is preferable since it allows the quantification of model fitting in multiple ways [5]. Altogether, the different methods of factor analysis first extract a set a factors from a data set, which are ordered according to the proportion of the variance of the original data they explain. While a subset of factors is kept for further analysis, others are considered as either irrelevant or nonexistent (that is, reflect measurement error or noise) [6]. Rotation of the factor axes (dimensions) identified in the initial extraction is then conducted in order to obtain simple, reliable and interpretable factors [7].

Still, despite the established need and use of factor analysis methodologies, including in neurocognitive studies, the decision between different methods is often confusing and difficult for the “average second-language” researcher on statistical methodology, rendering it difficult to decide on the appropriate technique or even where to start. For instance, many investigations into the structure of individual differences theorize in terms of latent variables, but rely greatly on PCA when analyzing the data. In fact, this constitutes a debated area because PCA is a data reduction technique and not a latent variable one, and it is a formative model and not a reflective one (conceptualizing constructs as causally determined by the observations, not the other way around) [8].

This is of particular interest in cognitive ageing research, given that the researcher is the one who has the responsibility of analyzing the cognitive data measured via neurocognitive/psychological test batteries. Often, test variables must be grouped as reflective of the overall cognitive ability across cognitive domains. Particularly, decisions must be made attending to the two broad cognitive dimensions that have emerged across studies as sensitive to age-related effects: memory and general executive function [9–15]. In fact, evidence on age-associated memory and executive cognitive changes are so well-established that they might be considered the baseline against which other variables are analyzed [14]. In this context, and as recently reported, overall there are many studies lacking on transparency on the decisions on methodology, often lacking on adequate reporting in the design, conduct and analysis of the experiments [16].

In this line, herein the goal was to analyze the factor structure of neuropsychological measures, both with exploratory and confirmatory methods, and also to provide relevant support for methodological decisions.

## Material and Methods

### Participants

Participants (n = 1051) were randomly selected from the Guimarães and Vizela local area health authority registries. The cohort was representative of the general Portuguese population with respect to age and gender. All participants still resided in the community (community-dwellers), the majority was retired (n = 763, females 51.8%) and in the medium socio-economic stratum (61.6%, females 47.3%; Graffar measure [17]). Sample characteristics are presented in Table 1.

**Table 1:** Sample characterization

		Females	Males	Total
Gender		560 (53.3%)	491 (46.7%)	1051
Age	[50–59] years	141 (52.8%)	126 (47.2%)	267 (25.4%)
	[60–69] years	176 (53.7%)	152 (46.3%)	328 (31.2%)
	70 or more years	243 (53.3%)	213 (46.7%)	456 (43.4%)
School years	3 or less years	259 (71%)	106 (29%)	365 (34.7%)
	4 years	246 (47.4%)	273 (52.6%)	519 (49.4%)
	5 or more years	55 (32.9%)	112 (67.1%)	167 (15.9%)

The study was conducted in accordance with the Declaration of Helsinki (59th Amendment) and was approved by the national ethical committee (Comissão Nacional de Protecção de Dados) and by the local ethics review boards (Hospital Escola Braga, Braga; Centro Hospitalar do Alto Ave, Guimarães; and Unidade Local de Saúde do Alto Minho, Viana-do-Castelo/Ponte-de-Lima). Potential participants were explained the study goals and the neurocognitive evaluation. All volunteers provided written informed consent.

### Neurocognitive assessment

Tests were selected to provide cognitive (general cognitive status and executive and memory functions) profiles. A team of trained psychologists conducted the cognitive/psychological evaluations, constituted by the following instruments: the Mini-mental state examination (MMSE) [18], which is the most widely used cognitive mental status-screening test and assesses orientation, word recall, attention and calculation, language and visual-construction abilities; the Digit Span test [19–22], used as a measure of short-term memory, working memory and attention; the Stroop test [23] to test for the ability to resist to interference and to assess cognitive flexibility and inhibitory control; the Selective reminding test (SRT) [24], to evaluate verbal learning and memory through the parameters long-term storage (LTS), consistent-term retrieval (CLTR) and delayed recall; and the Controlled Oral Word Association Test (COWAT-FAS) [25], which is a measure of verbal fluency. All neurocognitive test scores were converted into *z* scores to express all variables in the same scale.

### Exclusion criteria

Participants that met the established MMSE criteria for cognitive impairment were excluded from the sample (n = 3, 0.3%) [26] Furthermore, following a very conservative approach, individuals with one (n = 31, 2.9%) or more (n = 336, 32.0%) missing values in the neuropsychological test battery were also excluded [filling the requirements for an appropriate strategy according to (Rubin, 1976)]. The remaining participants (n = 684) were equally allocated at random into two groups: one for EFA and PCA, to investigate the number of factors underlying the neuropsychological variables (group termed “EFA/PCA”); the second to test the “best fit” model via CFA (group termed “CFA”). The sample sizes were appropriate to conduct the described statistical procedures [27], and Stevens [28] recommendations were met (ranging from 5–20 participants per scale item). Replicability analysis was conducted via internal replication (splits a single data set into two samples via random assignment). The participants were randomly assigned into the two groups. This was a calibration and validation samples strategy. Both samples remained representative of the initial study population for all the socio-demographic measures considered, except regarding literacy rate (99.4%, able to read and write, in both groups).

Subsequently, participants were considered outliers if in any of the neurocognitive variables a *z*-score>|4| was obtained (n = 9 participants in both samples; representing 2.6% of each sample). A conservative approach was followed; to obtain variables with approximate normal distribution, skewness and kurtosis values were considered in this decision (skewness value < |3| and kurtosis < |8|), following Kline [29] reference values.

### Exploratory factor analysis (EFA) and Principal component analysis (PCA)

In EFA several factor analysis extraction methods are used, but some controversy is still observed. Whereas some argue for severely restricted use of components analysis in favor of a true factor analysis method, others argue that there is almost no difference between these, or even that the former is preferable (see references in favor of the different views in review, [30]). Briefly, as supported by Fabrigar and colleagues [31], maximum likelihood (ML) [32] and principal axis factoring (PAF) yield the best results. Specifically, if data are relatively normally distributed, ML is the best choice, while if the assumption of multivariate normality is “severely violated”, the use of principal factor methods is recommended. Despite significant controversy in the field over the equivalence between the factor analysis and PCA, PCA remains a highly popular technique and its use has been supported based on similarity in results between the techniques and gains in information [31].

Two main types of rotation are used: orthogonal and oblique, assuming that the factors are uncorrelated or correlated, respectively. Most of the rationale for rotating factors comes from Thurstone [33] and Cattell [34] for deciding on simple structure (a goal of rotation methodology if to measure a single construct) [35]. It is, therefore, important to note that if allowing for the possibility that an instrument measures multiple constructs, simple structure would not be the goal [36]. An orthogonal rotation is specifled by a rotation matrix, the rows stand for the original factors, the columns for the new (rotated) factors and at their intersection the cosine of the angle between the original axis and the new one is formed. Four rotation methods are listed by Gorsuch [37]. The most popular orthogonal rotation technique is varimax [38]; where, a linear combination is desired so that the variance of the loadings is maximized. In oblique rotations the new axes can take any position in the factor space (both orthogonal and oblique rotations are performed in a “subspace” referred to as the factor space); however, the degree of correlation allowed among factors is normally small (this is because two highly correlated factors can be better interpreted as only one factor). Gorsuch [37] lists 15 different oblique rotation methods that, in general, are almost always interpreted by looking at the correlations between the rotated axis and the original variables and are interpreted as loadings. From an oblique rotation two different matrices are obtained that can (and should both) be used for interpretation: the structure matrix, which holds the correlations between each variable and each factor (same as with orthogonal rotations); and the pattern matrix (factor loadings), which holds the beta weights to reproduce variable scores from factor scores. Among oblique rotations, direct oblimin [37, 39] is the more generally used method. This rotation allows for the definition of the magnitude of the correlation by the researcher. This can be particularly useful if there is theoretically-based knowledge concerning the degree of correlation between factors.

Given this information, for the present dataset it is thought that the more appropriate extraction method is ML with oblimin rotation. However, so to avoid *a priori* assumptions, here, three different dimensions/components extraction methods were tested: ML, PAF and PCA. For each method, two rotation methods were tested: orthogonal (varimax) and oblique (oblimin). In all methods all neurocognitive measures were considered for the initial analysis. In modeling, in the reflective models, the parameters SRT intrusions and COWAT F-A-S non-admissible were excluded due to very small initial communality values (.034 and .047, respectively), in both rotation methods models. Furthermore, for the variable SRT intrusions, the measures of sampling adequacy based on the anti-image correlation were < .5 (.337). For all other measures the anti-image correlation values were > .5. In the formative model, in both rotation methods, the variable SRT intrusions was excluded due to anti-image correlation < .5 (.337), followed by exclusion of MMSE due to similar and absolute loading < .4 in two separate components.

### Parallel analysis (PA)

In order to determine the number of factors to extract in EF and PC analyses, many criteria have been proposed [40, 41]. Among these, the parallel analysis (PA) method [42] is used to determine the threshold for significant components, variable loadings, and analytical statistics when decomposing a correlation matrix. Specifically, it results from a modification of the Cattell’s scree diagram to ease the component indeterminacy problem [43], and it can be used to statistically verify the number of factors extracted in EFA and/or PCA. For this, it requires that a set of random correlation matrices are generated using the same number of variables and participants as the experimental data used in the EFA and/or PCA procedure [41, 43]. PA can use different methodologies to determine (or “confirm”) the number of components to extract [44–46]. Some authors argue that the routine use of PA in multivariate ordination increases the confidence in the results, and reduces the subjective interpretation of supposedly objective methods, because it allows to determine which variable loadings are significant for each component, thus parsimoniously simplifying structure and reducing the analysis of noise [6].

Here, the optimal number of components factored was determined based on the original eigenvalues (raw data values ≥1; Kaiser criteria) and subsequently comparison with the 99 percentile, obtained using the O’Connor [47] procedure. For this, to confirm the number of factors extracted in EFA and/or PCA, PA (eigenvalue Monte Carlo simulation) multiple strategies were used including: PC/PAF/common factor analysis with random normal data generation and raw data permutation. The number of datasets considered was 2,000 for each strategy and the percentile considered was 99. Other approaches could be explored such as the Scree plot test proposed by Cattell [48] and the Velicer’s MAP test Minimum Average Partial [40]. In fact, the Kaiser’s eigenvalue-greater-than-one rule approach has deficiencies [49] and should be used with a confirmatory approach, which was the strategy here followed by using PA. PA is one of the procedures with increasing consensus among statisticians to typically yield the most fit to data solutions regarding the number of components/factors [47]. Furthermore, the Kaiser criteria and the Scree plot criteria are similar in the manner that both are “mechanical rules-of-thumb,” while PA is statistically based.

### Confirmatory factor analysis (CFA)

The most common estimation procedure in SEM is ML, which assumes multivariate normality (MVN) [50]. On this, failure to meet the assumption of MVN can lead to an overestimation of the chi-square statistic and may undermine the assumptions inherent in several ancillary fit measures; still, a mild departure from MVN is acceptable (see full references on MVN assumptions in review, [50]). However, in CFA, upon model estimates, model fit must be evaluated. Besides the mentioned chi-square goodness-of-fit test, other ancillary indices of global fit are used, including: goodness-of-fit index (GFI) and adjusted goodness-of-fit index (AGFI), comparative fit index (CFI), Tucker-Lewis index (TLI), root-mean-square error of approximation (RMSEA), and the standardized root-mean-square residual (SRMR).

CFA was applied using the ML estimation method. Fit statistics/indexes of the tested models without and with correlation errors were tested (respectively, Fit Model A and Fit Model B; the latter with correlations between the Stroop test measures). The correlation among errors were established according to modification indices (MI) higher than 11 (χ^2^_0.999; (1)_ = 10.83). Although, Arbuckle [51] suggests MI ≥ 4 (χ^2^_0.95; (1)_ = 3.84), here a MI ≥ 11 was used for a more conservative approach, balancing model saturation and goodness-of-fit measures. A way to roughly gauge the size of the MI is to compare the chi-square difference relative to the overall chi-square value (i.e., by what percentage will the chi-square be improved by the addition of the new parameter to the model). The MIs, should a variable be dropped or a path added, are estimated to derive the best indicators of latent variables prior to testing a structural model (this is part of the process together with factor loadings and unique variances). When modifications are made to the model after an initial analysis or multiple models are tested, different indexes should be used [52]. Fit Model A and B’s Cronbach α values were .802 and .897 for F1 and F2, respectively, representing good internal consistency. Reliability coefficients of these magnitudes were within the range acceptable for psychological instruments [53, 54]. Data imputation was performed using Bayesian imputation method into a single output file (based on Model B).

### Construct reliability and validity

The reliability of the individual items (cognitive/psychological test variables) was assessed through regression weights (for all items λ_ij_ ≥ 0.5, R^2^ ≥ 0.25; following the Hair, Anderson [55] criteria), and the reliability of the construct (CR) through the composite reliability measure [56] (for both factors CR_j_ ≥ 0.7; following the criteria).

$$CR_{j}=\frac{{\left(\sum_{i=1}^{k}\lambda_{ij}\right)}^{2}}{{\left(\sum_{i=1}^{k}\lambda_{ij}\right)}^{2}+\;\sum_{i=1}^{k}\varepsilon_{ij}}$$

where,

$$\begin{array}{l} \lambda \;\;=\;\;standardized\;\;regression\;\;weights\\ \varepsilon_{ij}=\;1-R_{ij}^{2}≅\;\;1-\lambda_{ij}^{2} \end{array}$$

For construct validity, different validity strategies were used: factorial (CFA and correlation between constructs); convergent [average variance extracted (AVE)]; and discriminant [comparison of the shared variance (squared correlation) between the constructs against the average of the AVEs for these; AVE_j_ ≥ squared correlation between factors, following Fornell and Larcker [56]].

$$AVE_{j}=\;\frac{\sum_{i=1}^{k}\lambda_{ij}^{2}}{\sum_{i=1}^{k}\lambda_{ij}^{2}+\sum_{i=1}^{k}\varepsilon_{ij}}$$

Two multiple linear regressions were performed, considering latent cognitive scores as dependent variables (after Bayesian imputation in a single output file and one completed dataset) and gender, age and school years (both as quantitative) as predictors.

### Statistical analysis

In sum, for the exploratory step (EFA/PCA), three extraction (ML, PAF and PC) and two rotation (orthogonal and oblique) methods were used. Parallel analysis (PA) was used to determine the same number of extracted factors (PC/PAF/common factor analysis with random normal data generation and raw data permutation). The obtained model was confirmed using CFA (ML method; tests of significance and goodness-of-fit measures: chi-square, CFI, GFI, TLI, RMSEA and SMRS). Construct reliability (CR, regression weights and composite reliability measure) and validity (factorial, convergent and discriminant) were determined. The SPSS package v20 (IBM SPSS Statistics) and AMOS statistical package v21 [51] were used to conduct all statistical analysis.

## Results

### Latent cognitive structure

**Fig 1:**
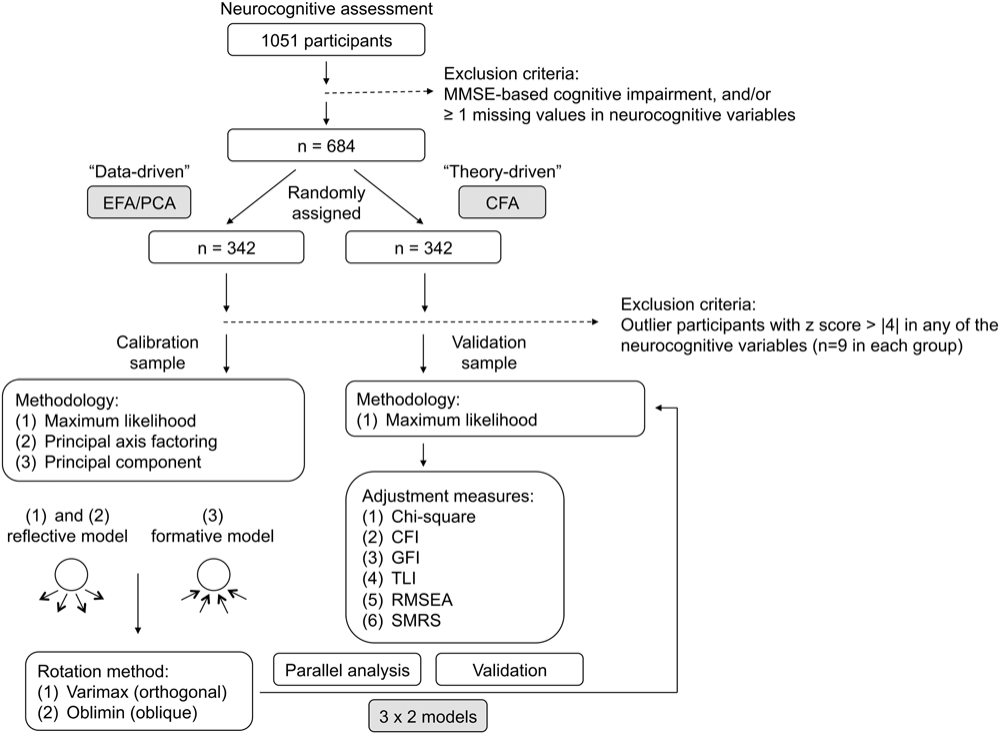
Factor analysis strategy for neurocognitive measures.

Data analysis was structured as described in Fig 1 and neurocognitive variables descriptive statistics for the “EFA/PCA” and “CFA” groups are presented in Tables 2 and 3. In general, variables were clustered in a similar manner across the three extraction methods (ML, PAF and PC) and the rotation methodologies (oblique and orthogonal) (Table 4). Specifically, for ML, for both rotation methods, a final ML solution with two factors (F1 and F2) was obtained. Sampling adequacy was met (KMO = .826) and there was a significant correlation between the variables (χ^2^(45) = 1341; p<0.001). Cronbach α values were .797 and .885 for F1 and F2, respectively, representing good internal consistency. As expected, variables saturated differently in each of the ML rotation solutions. Regarding PAF, a final solution with two factors (F1 and F2) was obtained in both rotation methods. Sampling adequacy, Bartlett’s sphericity and Cronbach α values were the same as those obtained in ML. Variables saturated differently in each of the PAF rotation solutions. Finally, in PCA a final solution with three components (F1, F2 and F3) was obtained in both rotation methods. Sampling adequacy was verified (KMO = .800) and a significant correlation between the variables was observed (χ^2^(45) = 1214; p<0.001). Cronbach α values were .777 and .885 for F1 and F2, respectively, representing good internal consistency; the F3 Cronbach α value could not be calculated due to the Factor being composed of only one variable. As expected, variables saturated differently in each of the solutions. Regarding the oblimin rotation method, for both ML and PAF the observed correlation between factors 1 and 2, for was .519, and for PC the correlation between components 1 and 2 was-.406, between components 1 and 3 was-.146, and between components 1 and 3 was .115.

**Table 2:** Descriptive statistics for the cognitive variables in the EFA and PCA.

	Raw Metrics							Z scores			
Neurocognitive variable (valid: n = 333)	Range	Min	Max	Mean	SD	Skewness	Kurtosis	Min	Max	Mean	SD
MMSE	13	17	30	27.1	2.43	-1.41	2.44	-3.84	1.16	0.03	0.93
Digits forward	15	0	15	7.6	2.08	0.65	1.06	-3.58	3.40	-0.04	0.97
Digits backward	10	0	10	4.0	1.79	0.74	0.49	-2.05	3.04	-0.03	0.91
Stroop words	94	11	105	62.7	18.15	-0.04	-0.29	-2.86	2.34	0.00	1.00
Stroop colors	64	18	82	47.9	12.37	0.26	0.19	-2.40	2.75	0.01	0.99
Stroop words/colors	64	0	64	28.9	11.37	0.71	0.56	-2.43	2.92	-0.01	0.95
SRT LTS	58	0	58	23.5	11.82	0.48	-0.03	-1.96	2.88	0.00	0.99
SRT CLTR	47	0	47	12.6	10.16	0.80	0.13	-1.23	3.31	-0.01	0.98
SRT delayed recall	12	0	12	5.3	2.16	0.08	0.24	-2.41	3.09	0.00	0.99
SRT intrusions	13	0	13	2.4	2.74	1.31	1.06	-0.84	3.72	-0.02	0.96
COWAT FAS admissible	49	0	49	17.1	9.69	0.78	0.47	-1.76	3.32	0.01	1.00
COWAT FAS non admissible	8	0	8	1.2	1.63	1.94	4.23	-0.69	3.69	-0.05	0.89

**Table 3:** Descriptive statistics for the cognitive variables in the CFA.

	Raw Metrics							Z scores			
Neurocognitive variable (valid: n = 333)	Range	Min	Max	Mean	SD	Skewness	Kurtosis	Min	Max	Mean	SD
MMSE	13	17	30	27.1	2.56	-1.40	2.31	-3.79	1.13	0.02	0.97
Digits forward	11	3	14	7.4	1.94	0.60	0.03	-2.27	3.45	0.00	1.01
Digits backward	10	0	10	3.8	1.77	0.85	0.98	-2.14	3.49	0.00	1.00
Stroop words	93	14	107	63.3	19.34	-0.09	-0.44	-2.56	2.26	0.00	1.00
Stroop colors	69	16	85	48.4	12.90	0.01	-0.22	-2.49	2.84	0.01	1.00
Stroop words/colors	64	5	69	28.9	11.35	0.64	0.51	-2.09	3.55	0.01	1.00
SRT LTS	59	0	59	25.3	12.50	0.24	-0.50	-2.00	2.71	0.02	1.00
SRT CLTR	57	0	57	14.2	11.68	0.84	0.41	-1.20	3.69	0.01	1.00
SRT delayed recall	12	0	12	5.6	2.37	0.11	-0.12	-2.34	2.70	0.02	1.00
SRT intrusions	14	0	14	2.6	3.14	1.38	1.37	-0.78	3.20	-0.03	0.89
COWAT FAS admissible	47	0	47	16.3	9.31	0.61	-0.02	-1.72	3.23	-0.01	0.98
COWAT FAS non admissible	8	0	8	1.2	1.63	1.73	2.71	-0.66	3.40	-0.06	0.83

**Table 4:** Comparison of the extraction and rotation methods in EFA (ML and PAF) and PC.

		Factor/Components loadings														
Extraction method	Rotation method	MMSE	Digits Forward	Digits Backward	Stroop Words	Stroop Colors	Stroop Words/colors	SRT LTS	SRT CLTR	SRT Delayed recall	COWAT FAS Admissible	COWAT FAS Non-admissible	Eigenvalues (after rotation)	Total variance (%)^b^	Cumulative variance (%)^c^	Cronbach α
**Factor 1**																
**ML**	**Oblique** ^**a**^	0.411	0.447	0.456	0.833	0.747	0.476	-0.007	-0.035	0.068	0.651	na	3.185	*	*	0.797
	**Orthogonal**	0.459	0.422	0.477	0.770	0.705	0.462	0.226	0.197	0.250	0.633	na	2.474	24.7	—	
**PAF**	**Oblique**	0.414	0.463	0.486	0.810	0.721	0.476	-0.008	-0.033	0.063	0.666	na	3.191	*	*	0.797
	**Orthogonal**	0.467	0.438	0.505	0.748	0.683	0.462	0.224	0.195	0.250	0.647	na	2.474	24.7	—	
**PC**	**Oblique**	na	0.586	0.495	0.829	0.775	0.593	0.008	-0.026	0.014	0.714	0.058	3.252	*	*	0.777
	**Orthogonal**	na	0.566	0.545	0.797	0.760	0.582	0.204	0.172	0.196	0.710	-0.014	2.782	27.8	—	
**Factor 2**																
**ML**	**Oblique** ^**a**^	0.253	-0.025	0.158	-0.113	-0.044	0.023	0.915	0.900	0.723	0.037	na	3.033	*	*	0.885
	**Orthogonal**	0.361	0.103	0.282	0.127	0.169	0.157	0.883	0.860	0.719	0.220	na	2.374	23.7	48.5	
**PAF**	**Oblique**	-0.271	0.024	-0.153	0.112	0.033	-0.021	-0.906	-0.886	-0.742	-0.031	na	3.041	*	*	0.885
	**Orthogonal**	0.379	0.108	0.286	0.120	0.172	0.155	0.874	0.847	0.735	0.219	na	2.375	23.8	48.5	
**PC**	**Oblique**	na	0.082	-0.176	0.036	-0.031	-0.020	-0.926	-0.920	-0.855	-0.089	-0.002	3.037	*	*	0.885
	**Orthogonal**	na	0.044	0.287	0.125	0.181	0.135	0.899	0.898	0.837	0.223	-0.037	2.518	25.2	53.0	
**Factor 3**																
**ML**	**Oblique** ^**a**^	na	na	na	na	na	na	na	na	na	na	na	na	na	na	na
	**Orthogonal**	na	na	na	na	na	na	na	na	na	na	na	na	na	na	
**PAF**	**Oblique**	na	na	na	na	na	na	na	na	na	na	na	na	na	na	na
	**Orthogonal**	na	na	na	na	na	na	na	na	na	na	na	na	na	na	
**PC**	**Oblique**	na	-0.143	-0.318	0.085	0.061	0.033	0.000	-0.001	0.004	0.102	0.957	1.155	*	*	na
	**Orthogonal**	na	-0.175	-0.356	0.034	0.010	-0.006	-0.045	-0.044	-0.038	0.053	0.950	1.069	10.7	63.7	

* = Cannot compute total variance after oblique rotation due to correlation of factors.

na = Not applicable (where applicable: for ML and PAF, only 2 factors were extracted and COWAT FAS non-admissible excluded due to very small initial communality values; for PC, MMSE excluded due to similar and absolute loading < .4 in two separate components and Cronbachα not computed for Factor 3 because only one variable).

^a^ Factor 2 was the first extracted factor.

^b^ Total variance accounted for after rotation.

^c^ Cumulative variance explained after rotation.

Since the goal was to explore a latent cognitive structure, the PC was excluded. Furthermore, because the two factors (dimensions) were expected to be correlated, the varimax solutions were also excluded. The ML and PAF results using oblimin rotation methodology were similar. This follows the literature indicating that although PAF is a better method to recover weak factors and that the ML estimator is asymptotically efficient, there is almost no evidence regarding which method should be preferred for different types of factor patterns and sample sizes [57]. Here, in accordance with the Kaiser’s criterion, the final PA solution yielded two factors in all methods, independently from whether the normally distributed random data generation or permutations of the raw data were used.

Based on these results, the ML oblimin two-factor solution was considered the “best fit” EFA solution, particularly taking into consideration that it is a reflective model and it is based on the same estimation method as the CFA methodology. Overall, this agrees with findings indicating that although PAF is preferred for population solutions with few indicators per factor and for overextraction, ML is preferred in cases of unequal loadings within factors and for underextraction [57], which more closely resembles our data. Furthermore, the results are also in agreement with the literature indicating that in contexts such as these it is better to conduct oblique rotations [58]. In general, when not knowing if the factors are or not related it is safer to conduct an oblimin rotation as i) there is not necessarily a reason to assume that the factors are independent, and ii) this rotation offers the advantage of estimating the factors’ correlations [58]. Based on the neurocognitive variables grouping, the obtained factors were termed “GENEXEC” (general and executive function; Factor 1, Table 4) and “MEM” (memory; Factor 2, Table 4). Specifically, “MEM” was composed of the variables SRT LTS, SRT CLTR and SRT delayed recall, and the variables MMSE, Digits forward, Digits backwards, Stroop words, Stroop colors, Stroop words/colors and COWAT FAS admissible saturated into the “GENEXEC” factor.

**Fig 2:**
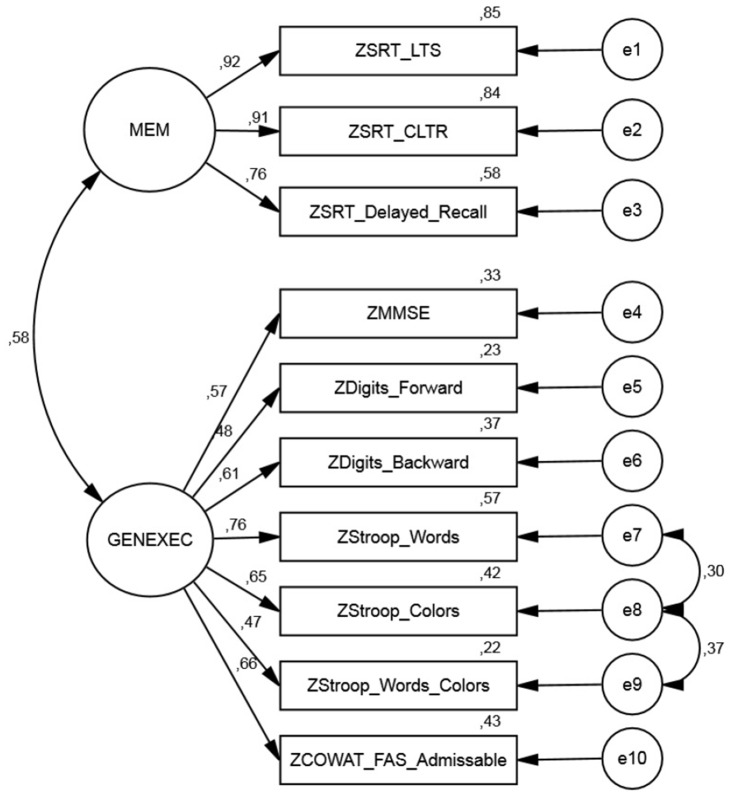
Confirmatory Factor Analysis.

Two CFA models were conducted: non-correlated and correlated error terms. For the non-correlated errors model (Model Fit A), the following tests of significance and goodness-of-fit measures were obtained: χ^2^(34) = 101.9; p<.001; χ^2^/df = 3.00 | CFI = .952 | GFI = .939 | TLI = .937 |RMSEA (HI90) = .078 (.095) | SMRS = .0519. Two error pairs were correlated according to the modification indexes with MI > 11: e7-e8 (MI 12.2) and e8-e9 (MI 33.1). Specifically, the Stroop words error term (e7) correlated with the Stroop colors error term (e8), which also correlated with the Stroop words/colors error term(e9). The CFA showed a satisfactory fit level for the correlated errors model (Model Fit B) with the following measures: χ^2^(32) = 44.9; p = .065; χ^2^/df = 1.40 | CFI = .991 | GFI = .974 | TLI = .974 | RMSEA (HI90) = .035 (.057) | SMRS = .0358. Comparing the overall significance of the initial model (A) with the model with correlated errors (B), the chi-square test difference shows that Model Fit B provides a significantly better fit than Model Fit A (Δχ^2^(2) = 57.0; p<.001). According to the goodness-of-fit measures, the model with correlated errors (Model Fit B) was considered the most favorable (Fig 2).

### Construct reliability and validity

Regarding the regression weights, for MEM for all items λ_i_ ≥ 0.5 [specifically, the smallest λ observed was .762 (R^2^ = .581) for SRT delayed recall], while for GENEXEC two regression weights presented values λ_i_< 0.5 [Stroop words/colors, λ = .467 (R^2^ = .218); Digits forward, λ = .484 (R^2^ = .234)]. Construct reliability was acceptable for the two factors (CR = .902 and .799 for MEM and GENEXEC, respectively) (Table 5). Regarding construct validity, for MEM the AVE = .755 and for GENEXEC the AVE = .368; that is, while the MEM factor presented acceptable scores (AVE ≥ 0.5), the GENEXEC factor did not fulfill the Hair, Anderson [55] criteria. It should be noted that the result is influenced by the items with low regression weights (Stroop words/colors and Digits forward); still, the items were kept in the analysis given that overall other reliability and adjustment measures criteria were fulfilled. Both factors, MEM and GENEXEC, presented AVE values higher than the squared correlation between factors (R^2^ = 0.335), meeting the [56] discriminant criteria (Table 5). Here, it should be noted that when the average variance extracted is below 0.5 it is hard to distinguish between variance due to construct and the variance due to error of measurement.

**Table 5:** Construct reliability and validity for the MEM and GENEXEC dimensions.

		Estimate	Sum	Sum (Squared)	Estimate (Squared)	1—Estimate (Squared)	Sum	Reliability	Validity
zSRT LTS	MEM	0.921			0.848	0.152			
zSRT CLTR	MEM	0.915			0.837	0.163			
zSRT delayed recall	MEM	0.762	2.598	6.750	0.581	0.419	0.734	0.902	0.755
zMMSE	GENEXEC	0.572			0.327	0.673			
zDigits forward	GENEXEC	0.484			0.234	0.766			
zDigits backward	GENEXEC	0.612			0.375	0.625			
zStroop words	GENEXEC	0.755			0.570	0.430			
zStroop colors	GENEXEC	0.648			0.420	0.580			
zStroop words colors	GENEXEC	0.467			0.218	0.782			
zCOWAT FAS admissable	GENEXEC	0.658	4.196	17.606	0.433	0.567	4.423	0.799	0.368

Two multiple linear regressions were performed, considering latent cognitive scores as dependent variables, and gender, age and school years as predictors (Table 6). Considering that age and school years have been proven to be important variables to cognitive ageing, here it was considered a good measure of results (model) validation. Both regressions were significant [for MEM: *F*_*(3*,*329)*_ = 24, p<.001; adjusted R^2^ = .172); for GENXEC: *F*_*(3*,*329)*_ = 48, p<.001; adjusted R^2^ = .299]; meaning that the three predictors explained 17.2% of the MEM latent score and 29.9% of the GENXEC latent score. Age and school years were significant predictors in both regression models. Age was negatively related with MEM (β = -0.283, p<.001) and GENEXEC (β = -0.260, p<.001) latent scores (controlling for gender and school years). School years was also significant for both regression models, although with higher impact on GENEXEC latent score (β = 0.383, p<.001) than MEM (β = 0.254, p<.001). Regarding gender, it was only a significant predictor for GENEXEC (β = 0.154, p<.001), with males scoring higher in this cognitive domain.

**Table 6:** Linear multiple regression models for MEM and GENEXEC latent scores.

	MEM						GENEXEC					
	B	S.E.	t	Beta	B 95% C.I.		B	S.E.	t	Beta	B 95% C.I.	
					L	U					L	U
Gender (male)	0.044	0.097	0.457	0.024	-0.146	0.234	0.141	0.044	3.244**	0.154	0.056	0.227
Age	-0.030	0.005	-5.476***	-0.283	-0.040	-0.019	-0.013	0.002	-5.467***	-0.260	-0.018	-0.009
School years	0.087	0.018	4.794***	0.254	0.051	0.122	0.064	0.008	7.857***	0.383	0.048	0.08

*p<.05;

**p<.01;

***p<.001.

Table 7 provides *z*-scores on the descriptive statistics, correlations and variance/covariance matrices for the variables in the model. The correlation matrices for the variables in the various datasets, split by the exploratory and confirmatory samples, are reported in Table 8.

**Table 7:** Descriptive statistics, correlations (below the diagonal), and variance/covariance (diagonal and above) matrices for the variables in the model.

Variable (z score)	1	2	3	4	5	6	7	8	9	10	11	12
MMSE	0.943	0.253	0.358	0.417	0.343	0.230	0.321	0.348	0.320	-0.073	0.340	-0.148
Digits forward	.259**	1.013	0.379	0.345	0.271	0.212	0.195	0.218	0.198	0.051	0.348	-0.001
Digits backward	.369**	.377**	0.997	0.438	0.367	0.300	0.322	0.351	0.355	-0.061	0.358	-0.085
Stroop words	.429**	.343**	.438**	1.001	0.651	0.401	0.343	0.362	0.373	-0.156	0.524	-0.094
Stroop colors	.354**	.270**	.368**	.652**	0.995	0.565	0.376	0.363	0.364	-0.043	0.412	-0.096
Stroop words/colors	.237**	.211**	.300**	.400**	.567**	1.000	0.284	0.235	0.247	-0.069	0.237	-0.120
SRT LTS	.331**	.194**	.324**	.344**	.378**	.284**	0.996	0.845	0.698	0.009	0.316	-0.085
SRT CLTR	.358**	.216**	.351**	.361**	.364**	.235**	.845**	1.003	0.684	-0.048	0.358	-0.108
SRT delayed recall	.331**	.197**	.357**	.374**	.367**	.248**	.702**	.686**	0.991	0.010	0.336	-0.114
SRT intrusions	-.084	.057	-.068	-.175**	-.049	-.077	.010	-.054	.012	0.796	-0.090	0.052
COWAT FAS admissible	.357**	.352**	.366**	.534**	.421**	.242**	.322**	.364**	.344**	-.103	0.962	-0.059
COWAT FAS non admissible	-.185**	-.002	-.103	-.114*	-.116*	-.145**	-.103	-.130*	-.139*	.071	-.073	0.684

n = 333 |

*p<.05;

**p<.01.

**Table 8:** Correlation matrix of the neurocognitive variables in the EFA/PC and CFA.

EFA/CP (CFA)												
Variable, z score	1	2	3	4	5	6	7	8	9	10	11	12
MMSE												
Digits forward	.240**											
	(.259**)											
Digits backward	.394**	.375**										
	(.369**)	(.377**)										
Stroop words	.375**	.313**	.344**									
	(.429**)	(.343**)	(.438**)									
Stroop colors	.320**	.242**	.326**	.624**								
	(.354**)	(.270**)	(.368**)	(.652**)								
Stroop words/colors	.279**	.196**	.258**	.306**	.496**							
	(.237**)	(.211**)	(.300**)	(.400**)	(.567**)							
SRT LTS	.415**	.173**	.345**	.294**	.309**	.250**						
	(.331**)	(.194**)	(.324**)	(.344**)	(.378**)	(.284**)						
SRT CLTR	.368**	.173**	.333**	.275**	.284**	.216**	.808**					
	(.358**)	(.216**)	(.351**)	(.361**)	(.364**)	(.235**)	(.845**)					
SRT delayed recall	.456**	.185**	.339**	.243**	.332**	.226**	.685**	.665**				
	(.331**)	(.197**)	(.357**)	(.374**)	(.367**)	(.248**)	(.702**)	(.686**)				
SRT intrusions	.035	.017	-.033	.012	.037	-.016	-.017	-.060	-.003			
	(-.084)	(.057)	(-.068)	(-.175**)	(-.049)	(-.077)	(.010)	(-.054)	(.012)			
COWAT FAS admissible	.435**	.354**	.444**	.540**	.397**	.265**	.340**	.312**	.291**	.105		
	(.357**)	(.352**)	(.366**)	(.534**)	(.421**)	(.242**)	(.322**)	(.364**)	(.344**)	(-.103)		
COWAT FAS non admissible	-.091	-.048	-.173**	-.060	-.088	-.062	-.091	-.084	-.071	.033	.004	
	(-.185**)	(-.002)	(-.103)	(-.114*)	(-.116*)	(-.145**)	(-.103)	(-.130*)	(-.139*)	(.071)	(-.073)	

*p<.05;

**p<.01

## Discussion

Here, we investigated the factor structure of neuropsychological measures. All cognitive evaluations were conducted using validated neurocognitive/psychological instruments (selected to evaluate underlying latent cognitive constructs). Taking into consideration that analysis and findings are based on the specific sample and measures (and may not necessarily generalize to other studies), summarily, among the explored EFA/PCA models, the EFA maximum likelihood strategy, using oblique rotation, proved to be the most adequate for the dataset yielding a two-factor solution: “MEM” (memory) and “GENEXEC” (general and executive function). Parallel analysis (PA) determined the same number of extracted factors. The obtained model was then confirmed using CFA in a separate sample from the same original cohort, with the results indicating a satisfactory fit level for a correlated errors model (Model Fit B). Largely, internal consistency and reliability coefficients were within an acceptable range.

Altogether, data analysis indicated that the obtained factors presented a moderate positive correlation between each other, which is not unexpected from a neurobiological/functional and/or structural point-of-view. For example, executive and memory functions both involve abilities such as verbal fluency, set shifting (“rule change”), attributed suppression (“interference”), temporal order, source memory, frequency estimation, working memory, free recall and recognition, that can be attributed to frontal lobe activity [59, 60]. However, it is also expected that the neurocognitive variables considered would load differently among the factors, following their inherent “design” purpose (or even why they were here selected for the cognitive assessment). Specifically, the variables MMSE, Digits forward, Digits backwards, Stroop words, Stroop colors, Stroop words/colors and COWAT FAS admissible saturated into the “GENEXEC” factor; while, “MEM” was composed of the variables SRT LTS, SRT CLTR and SRT delayed recall.

Executive function refers to a variety of higher cognitive processes that modulate and use information to produce behavior [19, 21], including initiation or intention of action, planning, working memory, and attention. Still results do remain interesting. For instance, the MMSE was not “clearly” in one of the two dimensions. As such, a confirmatory solution (CFA model) was tested where the variable MMSE was considered as a manifest variable of both cognitive dimensions; however, the regression weight was not significant (standardized regression weight: .105, p = .128; CFA model not shown; no significant differences between models based on chi-square difference statistics, p = .134). Regarding the SRT tasks, it is further interesting that the parameter SRT delayed recall presented the lowest score in its factor grouping, which might be rooted in the SRT tasks’ design characteristics (and, therefore, in the memory functions evoked by each). This means that while during the first five trials the participant has to find an efficient strategy to recall in a short time periods the presented words (“short-term storage”), in the sixth trial the participant must recall items now in “long-term storage” (SRT delayed recall). That is, the individual will be more efficient in remembering the words if throughout the initial trials he/she found a strategy to not only recall based on memory but also, based on some type of internal structure and/or association between the words and/or their meanings, find a “mnemonic” strategy that carries over to the delayed recall task.

Concerning the “GENEXEC” factor some considerations are warranted regarding the Stroop variables and their associated errors, which follow similar well-established findings in the literature. It is postulated that older subjects have fewer resources available to initiate efficient inhibitory processes in either divided attention tasks or if the task requires maintaining (“storing”) a large amount of information in working memory [61]. In this line, two separable control processes are deemed necessary in the Stroop task(s): goal maintenance (reflecting the ability to maintain the appropriate task set, such as, to respond “color” and ignore “word” across trials); and, response competition (reflecting the ease with which people can select between appropriate and inappropriate competing response tendencies) [62]. As such, a loss in goal maintenance can result in rapid-response errors in which the person simply responds to the word without any influence from the potentially competing color [62]. These aspects can justify the presence of Stroop-related errors observed in the CFA model. The efficiency at which the brain supports the ability to filter relevant stimuli from irrelevant noise can be gauged by “interference” effects in performance of classic selective attention/response conflict paradigms, such as the ones presented in the Stroop naming tasks [63]. These observed effects are considered to increase with age due to selective attention impairment, albeit some authors have suggested that this could also be a result of general slowing of information processing, since this effect disappears when processing speed is controlled with statistical analysis [64]. In model modifications a further consideration is warranted: the possibility that modifications fit chance characteristics of the sample data rather than the aspects that can be generalized to other samples [65]. As such, the model with correlated errors, here the most appropriate, may not be generalized to other datasets (and/or samples or population). In fact, the correlated errors CFA is not what emerged from the EFA; as such, the Model Fit B is not a “pure” confirmatory model, but instead it also contains an exploratory element (i.e., the correlation between the error terms).

As a measure of construct reliability and validity, multiple linear regressions were performed, considering latent cognitive scores as dependent variables and gender, age and school years as predictors (age and education are particularly well established measures in the literature in their relation with cognitive performance) (for example, [66]). Specifically, from the socio-demographic perspective, here the relation between the overall cognitive factors and education (higher education, better performance), age (cognitive performance decrease with age), and gender (males better performance in general executive tasks), follows with the literature. Furthermore, another relevant aspect is that human frontal lobes are particularly vulnerable to age-related deterioration and functional decline in ageing has been related to functions that largely depend on frontal regions [59]. For instance, normal ageing has been associated with difficulties suppressing irrelevant but highly activated responses [66]. This can be seen as a result of a decline in inhibitory abilities as well as in cognitive resources available to properly perform mental tasks [67]. Still, it should be mentioned that age sensitivity is attributed to functions also independent of frontal integrity [59]. Regarding gender, here, males presented a better performance in the GENEXEC cognitive domain and no significant association was detected between gender and MEM. In fact, the results reflect what still remains a matter of debate in the literature: the relation between “gender and executive function” and “gender and memory” in ageing studies. For this reason, gender itself should not yet be considered an adequate variable to attest any type of validity.

Of note, the factor structure of neuropsychological measures was already explored in a previous paper by the group [68]. As such, inherent good background knowledge on the properties and relationships between the test measures and the factors (MEM and GENEXEC) was already present. Nonetheless, a few observations should be addressed: the previous observations consisted of a smaller cohort, where the overall aim of the analysis (PCA) was to reduce information through a linear function and force the components not to be correlated (varimax). Here, the present study covers different exploratory methods to aggregate variables (formative: PCA; reflective: ML and PAF), as well as, through CFA, confirm the structural model found in a separate sample. Hence, the aim was also to capture a latent structure based on the cognitive/psychological tests and to confirm and validate the obtained latent structure. Additionally, a second order latent variable was also tested since a .58 correlation between MEM and GENEXEC was obtained, possibly suggesting a second order latent dimension. However, because only two first order latent dimensions were present and multicolinearity concerns should not be dismissed, this option was not further explored (data not shown). Also, in any case, the model suitably restricted is just a reparameterization of the correlated factor model. Still, this possibly indicates that other cognitive domains could be explored. In fact, preliminary data by the group indicates that measures of higher processing and/or related with cognitive reserve (such as measured by the Digit Symbol Substitution Test, DSST) may be of particular interest. Finally, although EFA is a widely-used technique it does contain inherent problems mainly at the level of replication; therefore, for instance, debate over the sample size, extraction and rotation techniques to use, number of factors to extract, and whether results of an EFA can be used in a “confirmatory” fashion, do remain [69]. As such, great care should be taken in any results generalization.

In summary, herein we have tried to exemplify that even taking into account the “vastness” of the field regarding factor analyses methodology and terminology, it is possible to follow a statistically “transparent” strategy to obtain the best possible model for the set of data at hand. Briefly, a reflective approach should be conducted to extract a latent construct, instead of a formative approach, which would be more appropriate to obtain an index. Still, although here results indicated that in order to capture latent structures in neurocognitive data, reflective models with *oblimin* rotation might prove the most adequate methodology, care should be taken on the measurement needs of each dataset, sample or population. As such results warrant further evaluation using a new dataset and a “true” method comparison analysis would benefit from a simulation study where varying conditions are introduced.
